# Assessing the impact of internet-based cognitive behavioral therapy on sexual dysfunction: A systematic review protocol

**DOI:** 10.1371/journal.pone.0324956

**Published:** 2025-06-05

**Authors:** Sanam Borji-Navan, Shahrbanoo Salehin

**Affiliations:** 1 Student Research Committee, School of Nursing and Midwifery, Shahroud University of Medical Sciences, Shahroud, Iran; 2 Sexual Health and Fertility Research Center, Shahroud University of Medical Sciences, Shahroud, Iran; University of South Australia, AUSTRALIA

## Abstract

**Introduction:**

Sexual dysfunction (SD), a prevalent health concern, can significantly impact an individual’s overall well-being and intimate relationships. Internet-based cognitive behavioral therapy (ICBT) has emerged as a promising and accessible intervention for addressing this issue. Therefore, this review aims to assess the impact of ICBT on SD among adults.

**Methods and analysis:**

Searches will be conducted on PubMed, Scopus, EMBASE, Web of Science (ISI), Trialserach.who.int, and ClinicalTrials.gov, as well as search engines like Google Scholar. This review will focus on randomized clinical trials evaluating the efficacy of ICBT in addressing SD. The initial search and subsequent removal of duplicate entries will be conducted by the principal investigator. Two independent reviewers will then perform study selection, data extraction, and two independent assessors will assess methodological quality using the Cochrane risk of bias assessment tool 2 (RoB 2). The final data synthesis strategy will be contingent upon the characteristics and availability of the included studies.

**PROSPERO registration number:**

CRD42023431255.

**Ethical code:**

IR.SHMU.REC.1403.079.

## Background

Sexual dysfunction (SD), a prevalent and distressing concern, exerts a substantial impact on individuals’ quality of life and interpersonal relationships. It encompasses a broad spectrum of difficulties, including challenges related to sexual desire, arousal, orgasm, and pain during sexual activity [1–4]. SD is a widespread issue, impacting roughly 43% of women and 31% of men [5]. SD has various causes, categorized as physical or psychological [6]. Physical causes include diseases like diabetes, heart conditions, obesity, hormonal issues, and certain medications or drug use [7,8]. Psychological factors, including stress, anxiety, depression, low self-esteem, and interpersonal challenges, such as marital disorders and societal and cultural pressures to conform to expectations regarding consummation of marriage, can also contribute significantly. Often, SD stems from a combination of both physical and psychological factors [9].

While various treatment modalities exist for SD, cognitive-behavioral therapy (CBT) is recognized as an efficacious approach due to its structured methodology and emphasis on modifying maladaptive cognitive and behavioral patterns [10–12].

The advent of technological advancements and increased internet accessibility has led to the emergence of internet-based cognitive-behavioral therapy (ICBT) as a potential alternative or adjunct to traditional in-person therapy [13–16]. ICBT presents several potential advantages, including enhanced accessibility, convenience, anonymity, and cost-effectiveness. However, the existing evidence base pertaining to the efficacy of ICBT in the treatment of SD remains fragmented and necessitates a systematic evaluation [17–19].

Zarski et al. explored the efficacy of internet- and mobile-based interventions (IMIs) in treating sexual dysfunction [17]. In contrast, our review focuses specifically on Internet-Based Cognitive Behavioral Therapy (ICBT), a distinct form of IMI. This distinction is crucial because ICBT has specific therapeutic components (e.g., cognitive restructuring, behavioral experiments) and delivery methods that may influence its effectiveness [20] and are not uniformly present across all IMIs studied by Zarski et al. Both our review and Zarski et al. (2022) assess sexual functioning. However, our review provides focusing on sexual quality of life as a secondary outcome. Our specific focus will allow us to synthesize evidence on how ICBT interventions uniquely impact individuals’ overall experience of their sexual lives, which is a critical aspect of sexual health beyond just functioning and satisfaction. This will provide a more holistic understanding of the benefits of ICBT. Zarski et al. provided a valuable synthesis of the evidence with a literature search ending in August 2021. However, the field of digital health interventions is rapidly evolving. Our search update has identified some new relevant studies published since that cutoff [21–25], highlighting the need for an updated systematic review to provide the most current evidence on ICBT for sexual dysfunction. A comprehensive literature search was conducted in PubMed, Scopus, EMBASE, Web of Science (ISI), PsycInfo, Trialsearch.who.int, and ClinicalTrials.gov, as well as prominent search engines such as Google Scholar. This search strategy differs from that of Zarski et al. which included Cochrane Central Register of Controlled Trials, PsycInfo, PubMed. Based on our knowledge, no systematic review has yet examined the effectiveness of ICBT for SD in adults in randomized controlled trials. This systematic review protocol aims to fill this gap by assessing the effectiveness of ICBT for SD by analyzing evidence from RCTs, specifically focusing on primary outcomes such as sexual function and secondary outcomes such as sexual satisfaction and quality of life.

## Objectives

### Primary outcomes

To examine the efficacy of ICBT for SD among adults.

### Secondary outcomes

To examine the efficacy of ICBT in improving sexual quality of life among adults.

## Methods and analysis

### Registry

This protocol has been approved by the Ethics Committee of the Shahroud University of Medical Sciences, Semnan, Iran (code number: IR.SHMU.REC.1403.079). This protocol was reported in accordance with the PRISMA-P guidelines [26,27] (S1 Checklist) and has been registered with PROSPERO under the registration number CRD42023431255. In the event of protocol amendments, all significant changes will be transparently documented (including the date of amendment and rationale) within the final published review, with concurrent tracking on the PROSPERO platform.

### Inclusion and exclusion criteria

The PICOS framework [28,29] will be employed to define the eligibility criteria for study selection, as detailed below (Table 1).

**Table 1:** PICOS framework.

	Property	Inclusion criteria	Exclusion criteria
**P**	**Population**	Adults with any type of SD (above 18 years).	Studies that do not include the adult age group.
**I**	**Intervention**	ICBT	Non-ICBT interventions (e.g., traditional in-person CBT, pharmacological interventions, non-psychological interventions, etc.).
**C**	**Comparison**	Placebo or any other intervention	–
**O**	**Outcome**	Primary outcome: sexual function Secondary outcomes: sexual satisfaction, quality of life and/or other related outcomes	Studies with other Outcomes.
**S**	**Study design**	Randomized controlled trials.	Systematic review, Meta-analysis, Qualitative studies, Letter to the editor, Conference papers, Case report and Case series studies, Protocols, Unpublished articles, Commentary, Grey literature, Meeting or conference abstract.

When encountering scholarly articles written in languages beyond the authors’ proficiency, machine translation technologies (e.g., Google Translate, AI-based tools) will be leveraged to facilitate interpretation and data extraction. No restrictions on language will be applied to articles published from July 1, 1990, to the Jan 28, 2025. This period was chosen because ICBT was developed in the late 1990s [30].

### Search methods and sources (search strategy)

In order to ensure the novelty of this research, a preliminary search was conducted on PROSPERO and the Cochrane Library to identify any existing or related systematic reviews. Furthermore, reference lists of identified systematic reviews will be examined to ensure a comprehensive identification of all relevant studies.

To ensure a capture of all relevant literature, we implemented a purposefully broad search strategy regardless of the specific inclusion criteria. Search terms were organized into conceptual categories (Intervention and Outcome), and pertinent keywords were identified for each concept, aligning with the study objectives and inclusion criteria.

A literature search will be conducted utilizing a multifaceted search strategy. Key terms and phrases will be identified through both structured and unstructured methodologies, leveraging controlled vocabularies (MeSH, EMTREE, ERIC) and free-text techniques (analysis of relevant articles, specialized texts, consultation with subject matter experts). The search will encompass all available languages.

The search strategy will be tailored to each specific database and search engine, ultimately combining keywords within concepts using the Boolean operator “OR” and linking concepts with “AND”. The initial search strategy, which can be further refined, for PubMed can be found in S2 File. The Polyglot Search Translator can be leveraged to enhance the efficiency of cross-database translation efforts [31].

The detailed search syntax for each database will be comprehensively documented in S1 Checklist. The principal investigator (SB) will execute searches across a range of databases, including but not limited to PubMed, Scopus, EMBASE, Web of Science (ISI), PsycInfo, Trialsearch.who.int, and ClinicalTrials.gov, as well as prominent search engines such as Google Scholar. Backward and forward citation searching methodologies will also be employed to enhance the research process.

### Study records

#### Data management.

Initially, citations will be uploaded to HubMeta [32], an online platform for managing systematic reviews and collaboration. Duplicates will be removed using HubMeta’s built-in feature. Full-text articles will be uploaded after title/abstract screening for full-text review.

#### Selection process.

Following this, a two-phase study selection process will be implemented. In the first phase, titles and abstracts will be comprehensively screened by two authors independently, and records that remain inaccessible in full-text format, even after three documented attempts to contact the corresponding authors and journal editor, will be excluded from further consideration. In the second phase, the full texts of the remaining records will undergo independent and evaluation by two reviewers, who will strictly adhere to pre-established inclusion and exclusion criteria. Any discrepancies arising between the reviewers will be resolved through a collaborative consensus-building process, or if necessary, by engaging a third-party arbitrator.

**Fig 1:**
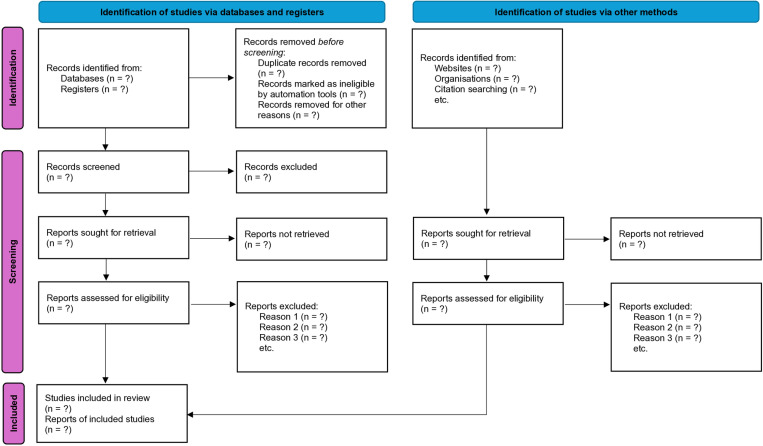
PRISMA flowchart.

A detailed and transparent report, encompassing all reviewed records and including a clear rationale for their inclusion or exclusion, will be compiled and presented in the standardized format of a PRISMA flowchart (Fig 1) [33]. Furthermore, at this juncture, it may be necessary to initiate communication with study authors to procure any missing data elements essential for comprehensive data synthesis.

### Data collection and analysis

Two independent researchers will then undertake a full-text review of each selected study, systematically extracting pertinent data elements using a standardized form designed by the research team. The standardized data extraction form will capture key information, including authorship, year of publication, country of study conduct, participant characteristics, study design, sample size, intervention and control group specifications, type and duration of intervention, measurement tools employed, and study outcomes. A standardized data collection form will be tested on five selected studies. Any necessary revisions to the form will be implemented following the pilot phase. The extracted data will subsequently be classified and organized. If required, study authors will be contacted to obtain any missing information deemed critical for robust data synthesis. In the event of any disagreement during the data extraction process, a consensus will be sought by involving the third author in a consultative capacity. Upon request from researchers, the data underlying our findings will be provided directly via email after the study is completed.

If sufficient data is available, we will analyze the existing evidence separately for female and male patients/clients to provide a comprehensive picture of the current state of ICBT for SD.

### Assessing study quality (risk of bias)

All included studies will undergo a methodological quality assessment conducted by two independent assessors using appropriate tools. This evaluation process will ensure the research’s methodological robustness and contribute to the overall validity and reliability of the findings.

After data extraction, two independent assessors will appraise the methodological quality of the included studies using the Cochrane risk of bias assessment tool 2 (RoB 2) [34].

The certainty of the evidence will be evaluated using the Grading of Recommendations Assessment, Development and Evaluation (GRADE) methodology [35]. Two independent assessors will assess the quality of evidence from studies contributing data to pre-specified outcomes using five GRADE considerations (risk of bias, consistency, imprecision, indirectness, and publication bias).

## Data synthesis and analysis

The data synthesis strategy will be contingent upon the nature and availability of included studies. A meta-analysis will be conducted if feasible to provide a quantitative synthesis of findings. Alternatively, a descriptive analysis will be employed to characterize and summarize key study characteristics and results.

A comprehensive qualitative synthesis will be performed, encapsulating key characteristics of included studies, interventions employed, outcomes assessed, and reported results. A systematic cross-study comparison will be undertaken, considering study design, participant demographics, intervention specifics, and evaluated outcomes. These synthesized findings will be interpreted within the context of the original research question and objectives, emphasizing identification of patterns, consistencies, and any notable discrepancies across studies.

Where applicable, a meta-analysis will incorporate studies based on the similarity of their outcome measures. Effect sizes (e.g., mean differences, standardized mean differences) along with their corresponding 95% confidence intervals will be computed for continuous outcomes. The choice between fixed-effect and random-effects models will be made following a comprehensive evaluation of the included studies. Heterogeneity will be assessed using the I² statistic, with values exceeding 50% signifying substantial heterogeneity. Subgroup analyses or meta-regression may be utilized to investigate potential sources of heterogeneity, such as variations in participant characteristics or intervention parameters. Sensitivity analyses will evaluate the robustness of findings by, for instance, excluding studies with high risk of bias or outliers.

Combined effect estimates with confidence intervals will be presented for each outcome, providing a comprehensive overview of intervention impact. Findings will be interpreted within the framework of clinical significance and relevance, considering implications for practice and future research. Potential limitations of the synthesis, including heterogeneity and risk of bias, will be transparently acknowledged and discussed to ensure methodological rigor.

## Discussion

The utilization of ICBT in addressing SD represents a promising and innovative avenue in the management of this prevalent and often distressing condition. This approach leverages technology to enhance therapeutic interventions, aiming to improve treatment accessibility, reduce stigma, and optimize outcomes for individuals experiencing SD.

This study will systematically review the effectiveness of ICBT for SD. It will assess clinical outcomes, explore the integration of technology into mental health care, and examine treatment adherence. By synthesizing existing evidence, this review aims to provide a clear understanding of ICBT for SD and identify areas for future research, ultimately contributing to more effective and accessible treatment options.

ICBT has the potential to revolutionize the treatment of SD, providing a more accessible, tailored, and impactful therapeutic option. However, it is imperative to address ethical considerations, rigorously evaluate the efficacy of these digital interventions, and ensure equitable access to maximize their benefits and facilitate their seamless integration into mainstream mental health care.

## Supporting information

S1 Checklist PRISMA-P 2015 checklist. (DOCX)

S2 File Search strategy. (DOCX)
