# Supplementary material for: Assessing the impact of internet-based cognitive behavioral therapy on sexual dysfunction: A systematic review protocol
Source: PLoS One. 2025 Jun 5;20(6):e0324956. doi: 10.1371/journal.pone.0324956 (PMC12140234; doi:10.1371/journal.pone.0324956)
Supplement: S2 File — (DOCX) [file pone.0324956.s002.docx]

**S2 File. Search strategy** (Terms used in the search strategy in PubMed database).

| Number | Search terms | | |
| --- | --- | --- | --- |
|  | Concept | | Syntax |
| #1 | **I** | Internet Based Cognitive Behavioral Therapy (ICBT) | "ICBT"[tiab] OR (("Internet-based"[tiab] OR "Internet based"[tiab] OR "Internet-delivered"[tiab] OR "Internet delivered"[tiab] OR Internet[tiab] OR “Web based”[tiab] OR Web-based[tiab] OR Webbased[tiab] OR "Online-based"[tiab] OR "Online based"[tiab] OR Online[tiab] OR digital[tiab] OR "Social Media"[tiab] OR “social network*”[tiab] OR computer*[tiab] OR on-line[tiab] OR Electronic[tiab] OR Virtual[tiab] OR Remote[tiab] OR Distance[tiab] OR Mobile[tiab] OR Game-Based[tiab] OR “Game Based”[tiab] OR Gamif*[tiab] OR Telemedicine[tiab] OR Tele-medicine[tiab] OR “Tele medicine”[tiab] OR teletherapy[tiab] OR tele-therapy[tiab] OR “tele therapy”[tiab] OR Teleeducat*[tiab] OR Telehealth[tiab] OR tele-health[tiab] OR “tele intervention*”[tiab] OR Telecourse*[tiab] OR mHealth[tiab] OR m-health[tiab] OR etherapy[tiab] OR e-therapy[tiab] OR "E Therap*"[tiab] OR eHealth[tiab] OR e-health[tiab] OR e-learning[tiab] OR eLearning[tiab] OR e-education[tiab] OR m-learning[tiab] OR mLearning[tiab] OR Mobile[tiab] OR "Telephone-based"[tiab] OR "Telephone based"[tiab] OR "Telephone*"[tiab]) AND ("cognitive behavioral therap*"[tiab] OR "cognitive behavioural therap*"[tiab] OR CBT[tiab])) OR (("Cognitive behavioral therap*"[tiab] OR "cognitive behavioural therap*"[tiab] OR CBT[tiab]) AND (app[tiab] OR apps[tiab] OR application*[tiab] OR "Text Messag*"[tiab] OR Texting[tiab] OR SMS[tiab])) |
| #2 | **O** | Sexual Dysfunction | "Sexual Dysfunction*"[tiab] OR “Sex Disorder*”[tiab] OR “Sexual Disorder*”[tiab] OR "Dyspareunia"[tiab] OR “Ejaculatory Dysfunction*”[tiab] OR “Ejaculation Dysfunction*”[tiab] OR “Premature Ejaculation*”[tiab] OR “Retrograde Ejaculation*”[tiab] OR “Erectile Dysfunction*”[tiab] OR "ED"[tiab] OR Impotence[tiab] OR Vaginismus[tiab] OR Anejaculation[tiab] OR “Delayed Ejaculation”[tiab] OR “Ejaculatory Incompetence*”[tiab] OR “Ejaculatio Praecox*”[tiab] OR “Male Impotence”[tiab] OR “Sexual Impotence”[tiab] OR “Psychosexual Disorder*”[tiab] OR “Psychosexual Dysfunction*”[tiab] OR Frigidity[tiab] OR “Hypoactive Sexual Desire Disorder*”[tiab] OR “Orgasmic Disorder*”[tiab] OR “Sexual Arousal Disorder*”[tiab] OR “Arousal Disorder*”[tiab] OR “Sexual Aversion Disorder*”[tiab] OR “Aversion Disorder*”[tiab] OR “Gender Identity Disorder*”[tiab] OR “Identity Disorder*”[tiab] OR “Sexual Addiction*”[tiab] OR “Sexual Compulsiveness*”[tiab] OR “Sexual Obsession*”[tiab] OR “Sexual Behavior*”[tiab] OR “sexual behaviour”[tiab] OR “Sex Behavior”[tiab] OR “sex behaviour”[tiab] OR “Sex Addiction*”[tiab] OR “Hypersexuality Disorder*”[tiab] OR “Sexual and Gender Disorder*”[tiab] OR “Gender Dysphoria”[tiab] OR “Sexual Health”[tiab] OR “sexual well being”[tiab] OR “sexual wellbeing”[tiab] OR “sexual well-being”[tiab] OR “sexual wellness”[tiab] OR Libido[tiab] OR Arousal[tiab] OR "Sexual Arousal"[tiab] OR "Orgasm"[tiab] OR "Vaginal Dryness"[tiab] OR "Sexual Excitement"[tiab] OR Orgasm*[tiab] OR "Sexual Satisfaction*"[tiab] OR "Sexual Gratification*"[tiab] |
| #3 | #1 AND #2 | | |
| #4 | 1990/01/01:2025/02/01[dp] | | |
| #5 | #3 AND #4 | | |
